# The paraty artisanal fishery (southeastern Brazilian coast): ethnoecology and management of a social-ecological system (SES)

**DOI:** 10.1186/1746-4269-8-22

**Published:** 2012-06-27

**Authors:** Alpina Begossi, Svetlana Salyvonchyk, Vinicius Nora, Priscila F Lopes, Renato AM Silvano

**Affiliations:** 1 UNICAMP: CAPESCA, LEPAC (Paraty) and CMU, CP 6023 Campinas, Brazil; 2 ECOMAR/UNISANTA, Rua Oswaldo Cruz, 277, Santos, SP, CEP 11045-907, Brazil; 3 FIFO (Fisheries and Food Institute), ECOMAR/UNISANTA, Santos, Brazil; 4 Institute for Nature Management, National Academy of Sciences of Belarus, 10 Fr. Skaryna Street, Minsk, 220114 Minsk, Belarus; 5 Dept. Botany, Zoology and Ecology, Federal University of Rio Grande do Norte, Natal, RN 59078-900, Brazil; 6 Departamento de Ecologia, UFRGS, Porto Alegre, RS, Caixa Postal 15007, 91501-970, Brazil

## Abstract

This study intends to give recommendations to the management of Paraty fishery in Brazil through an interplay of local and scientific knowledge. In particular, the objectives are the following: 1) to describe the Paraty fishery; 2) to compare the fishermen’s local ecological knowledge with recorded fish landings and previous studies in Paraty; 3) to combine the data on local fishing and on local/Caiçara livelihoods with the SES (social-ecological systems) Model. The methods include a systematic survey of fishing in Tarituba and Praia Grande, which are located in the northern end and the central part of the Paraty municipality, respectively. For four days each month, systematic data on catches at landing points were collected, as well as macroscopic gonad analysis data for the fishes *Centropomus parallelus* and *C. undecimalis* (snook, robalo), *Epinephelus marginatus* (grouper, garoupa), *Scomberomorus cavalla* (King mackerel, cavala), and *Lutjanus synagris* (Lane snapper, vermelho). Spring and summer are important seasons during which some species reproduce, and the integration of fishing periods for some target species could assist in fishing management through the use of closed seasons. Fishermen could obtain complementary earnings from tourism and from the “*defeso* system” (closed season including a salary payment) to conserve fishing stocks. The SES model facilitates an understanding of the historical context of fishing, its economic importance for local livelihoods, the constraints from conservation measures that affect fishermen, and the management processes that already exist, such as the *defeso*. If used to integrate fishing with complementary activities (tourism), such a system could improve the responsibility of fishermen regarding the conservation of fish stocks.

## Background

Studies on artisanal or small-scale fisheries have included concerns regarding fishery management, leading to the development of mechanisms and concepts to describe local fishery knowledge, management and governance. Many authors have studied these topics using the following approaches: long-term analyses of the system of lobster catches, including the ‘boom and bust’ of lobster production; the fishermen’s perceptions of the resource; the fishermen’s property rights system; and the development of governance and co-management systems [1,2]. Studies addressing the diversity of fishing contexts have demonstrated the importance of local fishing rights in management processes [3]. In addition, Johannes (1988, 2002), among others, has made explicit propositions for implementing local ecological knowledge (LEK) as a tool for managing fisheries and to aid in the development of community-based management in Oceania [4,5]. Recently, Huntington (2011) has emphasized the importance of the collaborative process between researchers and indigenous peoples in fishery management [6]. In this sense, LEK can be very useful when used in tandem with or complementary to scientific knowledge.

Other concepts that have been studied and used in fishery management include the property rights systems in diverse fishing communities from different geographical areas, the fisherman’s local knowledge and governance and the ecological concept of resilience applied to adaptive management [7-10]. Conflicts over resources and resource management, including an understanding of institutions and local rules, have modeled situations of cooperation, reciprocity, and governance. In addition, specific rules regarding management and its embedding insertion into local contexts and institutions have been modeled and proven useful for fishery management applications [11,12]. In Chile, the concept of path dependency (which establishes a relationship between future choices and previous decisions, such as in a stabilized feedback mechanism) has been used to analyze the management of coastal fisheries and their management processes [13]. Path dependency may be appropriate for application to Brazilian coastal fisheries because Brazil and Chile are similar in their development, history, and some of the coastal interactions of their native populations, although Chile’s coastal fisheries are significantly more productive [14]. Feedback based on previous fishing experiences is a mechanism employed by fishermen in decision-making processes to influence the probability of a successful fishing trip, such as in decisions about the locations to fish [15], fishermen interactions concerning fishing spots, and catches [16].

Considering the existence of a dialectic [17] interchange between different systems of knowledge, referred to here as local and scientific, the mechanisms by which these two systems interact can be assumed to have overwhelming importance in the management of resources that are used locally by native populations. This assumption is based on the following: a) the necessity of inter-culture communication and the importance of applying ongoing (or traditional) local rules to address local management; b) the understanding of the process that influences the results of the interactions between fishermen and researchers, for example, that has previously been illustrated in the literature [4,5,18]. Thus, studies [19-21] have developed analyses and recommendations regarding the interchange between these two systems of knowledge, particularly for ecology and local ecological knowledge. These studies compare both systems to demonstrate their importance and shortcomings for the management of small-scale fisheries.

In Brazil, the literature has analyzed the interaction between local and scientific knowledge, including studies of folk knowledge and folk systematics, as well as the following: local rules for fishing activities [21-23]; ethnoecological, ecological and economic characteristics of coastal small-scale fisheries [24,25]; local knowledge regarding important target fish species [26,27]; and management processes, including variables and patterns associated with the resilience of the fishery management [28]. The history, local knowledge, culture and management of the local indigenous populations of the southern coast of Brazil, the *Caiçaras* (which are currently mostly coastal artisanal fishermen), are also important for linking local activities to fishing arrangements in other scales and contexts, such as livelihoods and markets [29-31].

Fisheries are complex and highly unpredictable [32,33]. Arrangement tools are necessary for understanding this complexity and evaluating their ecological-economic characteristics and demands. One of these tools is the SES (social-ecological system) model [34], which decomposes sets of variables (such as users, resources, and governance) and facilitates the analysis of complex processes and systems.

In addition to describing our case study of a one-year fishery landing in a tropical coast in southeastern Brazil, located in Paraty in the state of Rio de Janeiro, the objective of this study is to recommend management mechanisms for this fishery based on past and present information. For these analyses, we use the SES suggestive model to perform a discrimination or decomposition of variables to analyze the Paraty fishery.

Hence, this study has interconnected objectives with the common goal of understanding a social-ecological system, the Paraty fishery, during the years 2009–2011, based on data collection regarding artisanal fishery landings and interviews. No baseline is found for those coastal areas in Brazil regarding the production and catches in fisheries. The specific objectives are as follows:

1. To describe the Paraty fishery, including its diversity of catches, production, principal species, and fishing areas or spots utilized (here we consider fishing spots as parts of fishing areas)

2. To compare interview data to data obtained at landing points.

3. To compare and analyze landings with information about local ecological knowledge gathered in previous studies in Paraty [35].

4. To combine the data on local fishing, local livelihoods, and property rights, among others, for the Paraty coast and the Caiçara system from this and other studies [24, 25, 35]. These data can then be used to develop recommendations and mechanisms to manage the Paraty fishery and to include fishermen in this objective. To this end, objectives 1, 2, and 3 are connected and analyzed with the SES model (social ecological systems) [34].

## Study site and methods

**Figure 1:**
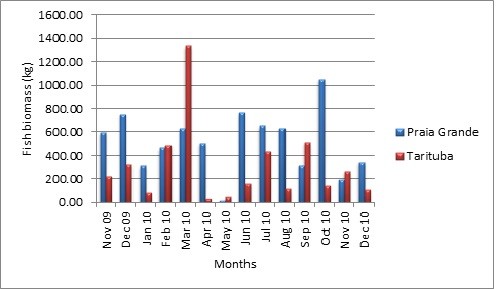
**Production of Paraty fishery (biomass of fish caught) at the landing points of Praia Grande and Tarituba (n = 360 trips).** Cold season from June to August, hot season from December to February.

**Figure 2:**
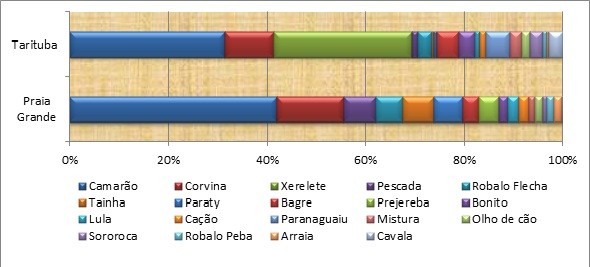
**Relative production (percentage of total biomass caught) per fish (folk names) of the artisanal fishery in Paraty in the landing points of Praia Grande and Tarituba (2009–2010) (n = 360 trips).** Some scientific names are available in Table 2, Additional file 1.

This study includes a systematic survey of fishing in Tarituba (the northern end of the Paraty municipality) and Praia Grande (the central part of the Paraty municipality). The southern end of the Paraty municipality is represented by the community of Trindade, a tourism-heavy site where fishermen use the cerco flutuante, an immobile floating trap net owned by some individuals. The corresponding preliminary results are published by Begossi [36]. This fishing differs from the active and independent style of fishing involving nets and hooks and lines used by the fishermen of Tarituba and Praia Grande in Paraty (Figures 1 and 2: Additional file 1).

The municipality of Paraty is located on the coast of Rio de Janeiro in between Brazil’s two major cities: Rio de Janeiro and São Paulo. Paraty receives tourists year-round from Brazil and elsewhere. For details, see (http://www.paraty.com.br) [25,35] (Figures 1 and 2: Additional file 1).

Small-scale fisheries in Paraty represent an important part of the local economy, particularly in sites such as Praia Grande and Tarituba. Fishing is a kinship activity in which families play a central role in learning, gear use, and commercial networks [35].

There are conflicts between small-scale fisheries and the industrial fisheries that enter the bay. Conflicts also exist between small-scale fisheries and protected areas that exclude fishermen from islands located inside the reserves. Details of those conflicts have been published previously [25,35].

This research was conducted in two steps. The first step entailed interviewing fishermen to gather information on the communities in Paraty that were associated with small-scale fishing (artisanal fishing). This method excluded fishermen who worked under contract in industrial fishing boats. Our aim was to access fishermen who depend on fishing for their livelihood and have strong interactions with a wide array or diversity of species, i.e., small-scale fishermen. LEK data were gathered in this first step, including a questionnaire with four sections: A) socio-economic information; B) fishing and LEK, such as fishing spots used, month of fish occurrence, gear used for each species, places where each species occur, and perceptions on the changes of fish abundance; C) use of natural resources and LEK, such as fish consumed and sold, fish used in local medicine, plants cultivated and used; D) conflicts in fishing activities, such as major problems, organizations, protected areas and interference with artisanal fishery. The results of this step were published [35], and they are used here for comparison and as a complement to the systematic observations at landing points. For this study, we use information related to the fishermen´s perceptions of production and abundance and the use of fishing spots (those based on the first step).

Fishing spots, fishing grounds, or, on a wider scale, fishing areas are specific sites at which fishermen search for specific species. Fishing spots are locally called ‘pesqueiros’ and often have rules of use [25,35]. Data on fishing spots used by the communities studied were gathered in interviews through the use of questionnaires in the first step of our study [35]. Fishing spots are located relatively close to the communities where fishermen live: technology limits fishermen´s possibilities to travel far away; moreover, there seems to exist an informal division over the use of the aquatic space, where fishermen from each community tend to use fishing spots close to their home communities [35].

The second step of the data collection was a systematic data collection on the main landing points of the aforementioned small-scale fisheries. To this end, we chose two points in the northern Paraty area: Praia Grande and Tarituba. Fishermen from Araújo Island, located in front of Praia Grande, also land with their catch at Praia Grande. At each of the landing points, a fish market or fish store received the marketable fish from landings and commercialized the fish (“Pescados Sinésio” in Praia Grande and “Peixaria Lara” in Tarituba). Over 13 months, from November 2009 to December 2010, one of the authors (VN) collected data for two days at each of the two landing points (four days of overall data collection per month). The data included catch weight per species, fishing spots used in the fishing trips, and an evaluation of the gonads (macroscopically) of some important target species mentioned in the interviews. We thus utilized two sources of data on the fishing spots used: the interviews in the first step and the systematic survey on landing in the second step of this study. We concentrated on the data collection on gonads and on the following target species: fat snook (robalo peba, *Centropomus parallelus*), common snook (robalo flecha, *C. undecimalis*), king mackerel (cavala, *Scomberomorus cavalla*), dusky grouper (garoupa, *Epinephelus marginatus*) and lane snapper (vermelho-ariocó, *Lutjanus synagris*). These species were chosen for gonad analysis to ensure the representation of estuarine, pelagic, and reef species, particularly threatened species such groupers and snappers [24,37].

The macroscopic gonad analysis involves observing visible eggs or sperm following a procedure already performed for other species, such as the common snook *C. undecimalis*[21], the dusky grouper *E. marginatus*[37], and the bluefish *Pomatomus saltatrix*[38]. In the macroscopic gonad analysis, the reproductive aspects were evaluated (presence or absence of eggs or sperm). We also measured the total length of the individuals of these species (two snooks, cavala, grouper and snapper). Data on reproduction and diet were also collected in the fish market mentioned above (landing points). Another fish market located in Perequê locality, near Tarituba, was included to increase our sample size for the reproductive observations of these target species.

Fish were identified by one of the authors (AB) at one of the landing points (Peixaria do Sinésio, Praia Grande, Paraty) using fish identification keys [39-45].

## Results

### Systematic sampling at the landing points of Praia Grande and Tarituba, Paraty, RJ (2009–2010)

The catches landed at the two artisanal fishery landing points in Paraty (Praia Grande and Tarituba) in the 2009–2010 sampling period totaled 11,471.89 kg (360 trips): 7,222.80 kg in Praia Grande (241 trips) and 4,249.09 kg in Tarituba (119 trips). The overall mean catch per trip was 31.9 kg: 30 kg/trip for Praia Grande and 36 kg/trip for Tarituba. We estimate that the mean daily production is 220.61 kg at both beaches after sampling the fishing for four days per month at Praia Grande and Tarituba (52 days of sampling). The annual production of the fisheries represented by Tarituba and Praia Grande is estimated at 529,586.40 kg, assuming 20 working days each month. No catch was obtained in 14 of the 360 trips.

Considering the landings and local markets, the Pescados Sinésio was responsible for 97% of the commercialization from the Praia Grande landings, and Peixaria Lara was responsible for 64% of the commercialization from the Tarituba landings. It is important to note that some fishermen sell directly to restaurants, tourists or the market ‘Sabor do Mar’ in the city of Paraty [35].

Figure 1 shows the production in kg per month from Praia Grande and Tarituba. The transitional months between the cold (dry) and hot (rainy) seasons were the most productive. November 2009 and 2010 were less productive months in Tarituba, but November 2009 was a productive month in Praia Grande. Differences in biomass and diversity were recorded for the December 2009 and 2010 catches (Additional file 1, Figure 1 and Table 1). These results illustrate the high variability of the productivity in the Paraty fisheries.

**Table 1:** The most caught species per month (biomass, kg) in the sampled landing points of Paraty, southeastern Brazilian coast (November 2009-December 2010)

		***Praia Grande***		***Tarituba***		***Total***	
**Month/Year**	**Place**	**Species**	**Production, kg**	**Species**	**Production, kg**	**Species**	**Production, kg**
Nov 09	1	Camarão 7 Barbas	193.50	Camarão 7 Barbas	66.50	Camarão 7 Barbas	260.00
	2	Robalo Flecha	154.10	Paranaguaiú	40.00	Robalo Flecha	192.50
Dec 09	1	Camarão 7 Barbas	280.00	Camarão 7 Barbas	73.00	Camarão 7 Barbas	353.00
	2	Corvina	91.80	Bagre	67.00	Corvina	108.30
Jan 10	1	Olho de Cão	45.00	Coroco	30.00	Olho de Cão	45.00
	2	Arraia Manteiga	44.00	Cavala	17.60	Arraia Manteiga	44.00
Feb 10	1	Camarão 7 Barbas	206.00	Camarão 7 Barbas	190.00	Camarão 7 Barbas	396.00
	2	Camarão Branco	112.35	Camarão Branco	169.00	Camarão Branco	281.35
Mar 10	1	Corvina	185.30	Xerelete	1,020.00	Xerelete	1,020.00
	2	Lula	153.00	Corvina	98.70	Corvina	284.00
Abr 10	1	Corvina	156.70	Bagre Amarelo	22.50	Corvina	159.00
	2	Parati	148.60	Corvina	2.30	Parati	148.60
May 10	1	Corvina	9.30	Corvina	31.00	Corvina	40.30
	2	Bagre Amarelo	8.00	Bagre Amarelo	8.00	Bagre Amarelo	16.00
Jun 10	1	Tainha	253.00	Camarão 7 Barbas	110.00	Camarão 7 Barbas	319.00
	2	Camarão 7 Barbas	209.00	Corvina	13.30	Tainha	253.00
Jul 10	1	Camarão 7 Barbas	176.90	Camarão 7 Barbas	129.00	Camarão 7 Barbas	305.90
	2	Pescada Branca	157.90	Galo	69.00	Pescada Branca	164.00
Aug 10	1	Camarão 7 Barbas	229.40	Corvina	30.50	Camarão 7 Barbas	259.40
	2	Corvina	100.00	Camarão 7 Barbas	30.00	Corvina	130.50
Sep 10	1	Camarão 7 Barbas	235.00	Camarão 7 Barbas	90.00	Camarão 7 Barbas	325.00
	2	Corvina	57.20	Paranaguaiú	84.00	Corvina	93.20
Oct 10	1	Camarão 7 Barbas	694.00	Camarão 7 Barbas	45.00	Camarão 7 Barbas	739.00
	2	CamarãoBranco	95.60	Sororoca	19.00	Camarão Branco	98.10
Nov 10	1	Camarão 7 Barbas	65.00	Camarão 7 Barbas	100.00	Camarão 7 Barbas	165.00
	2	Corvina	42.90	Corvina	45.00	Corvina	87.90
Dec 10	1	RobaloFlecha	122.40	RobaloFlecha	48.95	Robalo Flecha	171.35
	2	Camarão 7 Barbas	97.00	Corvina	15.90	Camarão 7 Barbas	97.00

**Figure 3:**
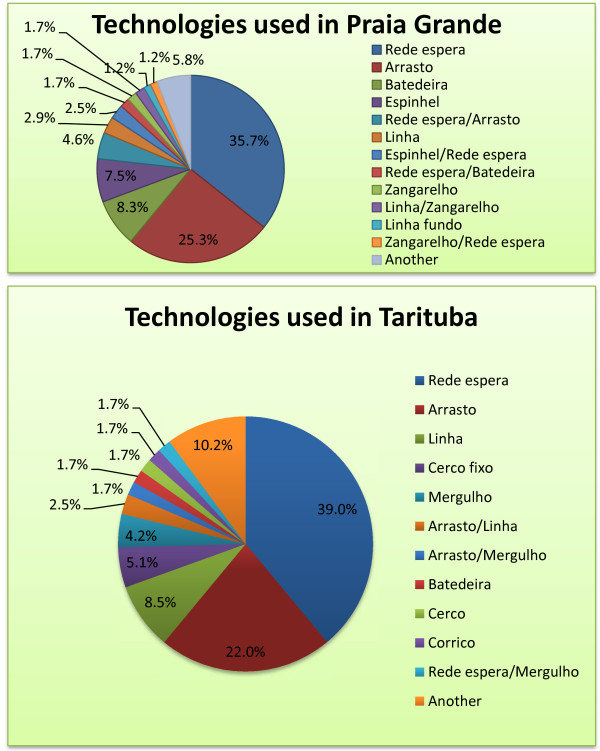
**a. Fishing gear and technologies used at the landing point of Praia Grande, Paraty, 2009–2010 (n = 241 trips).** Rede de espera = gillnets, arrasto = bottom trawl, espinhel = longline, linha = hand line, zangarelho = ripper jig.

Shrimp is the most important catch (for a list and identification, see the Additional file 1) at both Praia Grande and Tarituba, followed by fish such as sand drum (corvina, *Micropogonias furnieri*, and *Ophioscion punctatissimus*) and weakfish (pescada, *Cynoscion* spp., weakfish). Bluerunner (xerelete, *Caranx* spp.) is important in Tarituba (Figure 2). Fishing technologies are specific to the target species; set gillnets target many different fish species, and small trawlers (arrasto) target shrimp. Hooks and lines are also used in both artisanal communities for diverse species. ‘Cerco do robalo’ (for snook) is a diving technique used intensively in Tarituba (Figure 3a,b); this technique is also used by a few fishermen from Praia Grande and Perequê. Cerco do robalo involves catching snooks by using divers to encircle the fish with a net. This technique is controversial among local fishermen due to its alleged impact on fish resources (snook).

At Praia Grande, the production per landing point and per month demonstrates that, in terms of weight, shrimp is the most important catch in the summer and spring (eight months), sand drum is the most important catch in the autumn, and snook is important in the summer (Table 1). A similar pattern occurs at the Tarituba landing point, with the exception of the occurrence of bluerunner in March and Scombridae (king mackerel, cavala, *Scomberomorus cavalla* and Spanish mackerel, sororoca- *Scomberomorus brasiliensis*) and catfish in autumn (Table 1).

**Figure 4:**
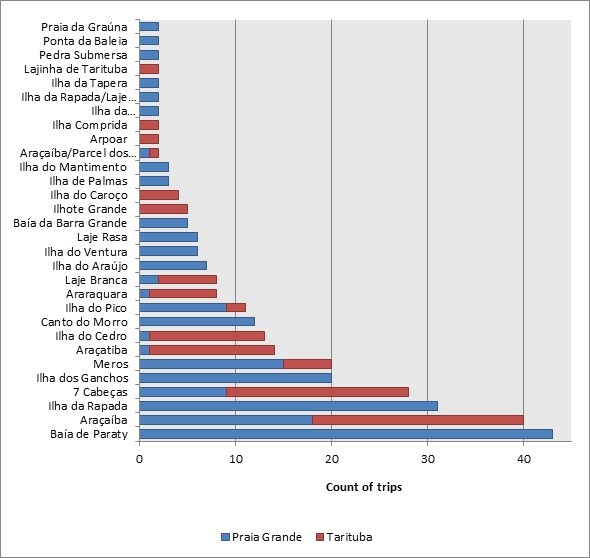
**Fishing spots used in the Paraty fishery from the landing points of Praia Grande and Tarituba (n = 360 trips).** It is unclear if Arcaíba and Araçatiba refer to the same location.

The primary fishing spots used by fishermen who land their catch at Praia Grande and Tarituba are shown in Figure 4. Six spots or locations account for most fishing trips (20 trips or more): Paraty Bay and the Rapada and Ganchos Islands for Praia Grande and Araçaíba, Meros and Sete Cabeças for both landing points. Two areas, Baia de Paraty and Ilha da Rapada, accounted for one-third of the fishing trips from the landing point of Praia Grande. Baia de Paraty includes diverse fishing spots because it is more like an area than a fishing spot in *stricto* sensu, whereas Ilha da Rapada includes only a few spots around the island. Half of the fishing trips from Tarituba included the fishing spots of Araçaíba, Cabeças, and Araçatiba, which account for approximately half of the total production (total production is 6,523.99 kg, Table 2). Cases in which two spots were used in the same fishing trip contribute 1,121.00 kg and are not included in the data in Table 2.

**Table 2:** Production of the most used spots in the sampled landing points of Paraty, southeastern Brazilian coast (November 2009- December 2010)

**Fishing spot**	**Location**	**Production, kg**	
Baía de Paraty	Paraty-RJ	0.75	1,421.45
	Pr. Grande	1,420.70	
Araçaíba^*a*^	Pr. Grande	531.05	925.75
	Tarituba	394.70	
Araçatiba	Pr. Grande	17.60	216.20
	Tarituba	198.60	
Ilha da Rapada	Pr. Grande	965.05	965.05
Sete Cabeças	Pr. Grande	117.25	678.85
	Tarituba	561.60	
Ilha dos Ganchos	Pr. Grande	439.69	439.69
Laje dos Ganchos	Pr. Grande	7.00	7.00
Meros	Pr. Grande	660.20	1,870.00
	Tarituba	1,209.80	
**Total**	**6,523.99**		

a We are not certain if Araçatiba is the same spot as Araçaíba.

### Reproduction of cavala, Snook and groupers

The estimates of the period of fish reproduction for important target species (two species of snook, dusky grouper and lane snapper), which were performed via a macroscopic analysis of the gonads, are shown in Table 3. Among the 62 individuals of king mackerel (*Scomberomorus cavala*) examined, only two females and thirteen males were mature, showing visible eggs or sperm, respectively, in the summer (December-March). No mature fish were found among the 83 groupers (*Epinephelus marginatus*) analyzed. Among the snooks, two species were analyzed: the robalo-flecha (*Centropomus undecimalis*) and robalo-peba (or peva) (*Centropomus parallelus*). Reproductive activity was observed for *C. undecimalis* in spring and summer and for *C. parallelus* in all seasons. The snapper *Lutjanus synagris* showed reproductive activity in the autumn and spring, particularly the latter (Table 3).

**Table 3:** Macroscopic gonad analyses for king mackerel, dusky-grouper, common snook, fat snook and lane snapper from recorded landings in Paraty in 2009–2011

		**Cavala King mackerel *S. cavalla***	**Garoupa Dusky grouper *E. marginatus***	**Robalo Flecha Common snook *C. undecimalis***	**Robalo Peba Fat snook *C. parallelus***	**Vermelho-ariocó Lane snapper *L. synagris***
**Count of fish with visible eggs**	Autumn			1	7	3
	Spring			5	7	8
	Summer	2		8	5	
	Winter				16	
	All seasons	2		14	35	11
**Count of fish with visible sperms**	Autumn	4		1	14	10
	Spring	1		20	6	9
	Summer	13		14		
	Winter				24	
	All seasons	18		35	44	19
**Count of fish with visible gonads**	Autumn	12	75	22	31	22
	Spring	11	3	7	20	19
	Summer	39		16	1	
	Winter		5	39	68	9
	All seasons	62	83	84	120	50
**Total count of fish**	Autumn	12	77	7	32	24
	Spring	11	3	39	15	20
	Summer	39	NS	24	6	
	Winter	NS	5	18	70	10
	All seasons	62	85	88	123	54

**Figure 5:**
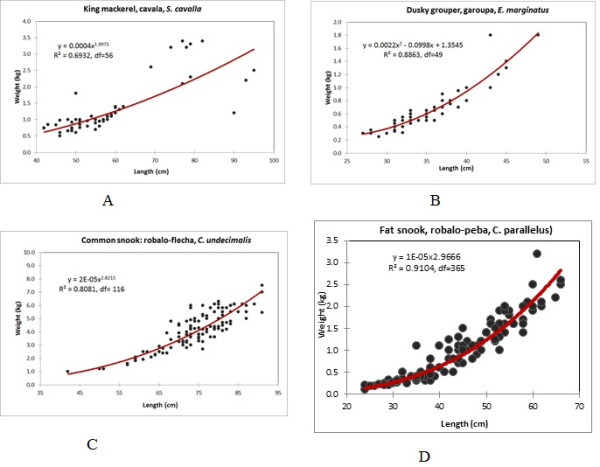
**A) weight and length of king mackerel (cavala, *Scomberomorus brasiliensis*)in samples from Paraty landings.** N = 56; **B)** weight and length of dusky grouper (garoupa, *Epinephelus marginatus*) samples from Paraty landings, N = 50; **C)** weight and length of common snook (robalo-flecha, *Centropomus undecimalis*) from samples from Paraty landings, N = 117; **D)** weight and length of fat snook (robalo-peba, *Centropomus parallelus*) from samples from Paraty landings, N = 111

With the exception of snook species, which have a more uniform size distribution, the fish were relatively small in length (total length). For example, king mackerel were 60 cm or less in length, and grouper were 50 cm or less in length (Figures 5a-d).

### Interviews with artisanal fishermen (n = 206 fishermen, January 2009)

In the interviews conducted during the previous year in Paraty (January 2009, 206 interviews), the Carangidae (xareu, *Caranx* sp.) and the Sciaenidae (sand drum, corvina, *M. furnieri*, among others) were most commonly cited as locally important in terms of production. At Praia Grande, shrimp, sand drum, weakfish, snook, mullet, and small shark were cited by fishermen as important, and snook was often mentioned at Tarituba [35].

Based on the interviews in Paraty (n = 206) concerning the catch from each fisherman’s most recent trip (“How much did you catch in your last fishing trip?”), the estimated catch by fishermen in interviews totaled 12,399.3 kg [35], averaging 74.2 kg per fisherman and 60.19 kg per trip (equivalent to 206 trips).

The most commercialized (sold) fish at Paraty, including the communities of Praia Grande (and Araújo island) and Tarituba, are snook, shrimp, weakfish, small shark, and snappers. The seafood most heavily consumed by fishermen from those areas were shrimp, sand drum, king mackerel (*S. cavala*), weakfish and snook. In the interviews, the fishing calendar (the periods when a certain fish is active) is as follows: snook, from October to February (summer); shrimp, from May to August (autumn-winter); and weakfish, from December to February and from May to July. For sand drum, there was no consensus on a specific period, although every village mentioned March as an important month. King mackerel was only mentioned in the interviews in Tarituba, where they estimated its season to be from December to February.

The fishing spots cited in interviews with fishermen were Ilha do Pico and Laje Branca at Praia Grande, Paraty Bay and Ilha do Araújo (the fishers from Ilha do Araújo land the fish at Praia Grande) at Ilha do Araújo, and Araçatiba and Araraquara at Tarituba. For all of Paraty, Meros, Ilha do Cedro, Paraty Bay, and the Rapada and Espia islands were the most cited fishing spots. The most productive spots are listed in Table 2 corresponding to the different interviews because the fishing spots cited at Praia Grande were not the most productive from the catch landed at Praia Grande (Table 2).

## Discussion

The production per month demonstrates the importance of shrimp as the principal catch landed in the local communities at both landing points, Praia Grande and Tarituba (Table 1). Snook (Centropomidae) occur in the summer, and weakfish and sand drum (Sciaenidae) are caught in the winter. Some fishery products were cited by fishermen in interviews as diminishing in supply [35]: sand drum, shrimp, snook, and weakfish, among others. These fish are important targets in the fisheries, as demonstrated by both interviews and landed production (Table 1, Additional file 1). We do not have access to temporal data due to a lack of a baseline; however, this issue should certainly be addressed because these species are commercially important. In fact, snook usually yields a higher fish price at the fish stores located at Praia Grande and Tarituba.

### Analysis of the resource (landings) and knowledge of the resource (interviews)

The information from interviews [35] is compared to the 2009–2010 landing data. The results of the catch landed from the systematic sampling and the results from the interviews addressed similar species, such as shrimp, sand drum (corvina, *M. furnieri,* among others,), bluerunner (xarelete, xaréu) and other species from the genus *Caranx*, along with weakfish (species of *Cynoscion*, among others, see Additional file 1 for details).

When comparing interviews and landings relative to the total production, we estimate approximately 30–36 kg per trip from the systematic landings and 60 kg per trip from the interviews. This difference can be explained in two ways: a) the fishermen overestimate the fishery production or b) our sampling covered days with poorer catch returns, causing an underestimation of the production in the Paraty fishery. Considering that returns from catches are highly variable (Figure 1 and Figure 3 in the Additional file 1), both hypotheses can be considered complementary and realistic. Other factor that difficult fishermen´s evaluations of fish availability is the fact that, as other hidden prey, fish are non-visible to humans, since they are located in the sea . By considering our two data sets to be complementary (direct observations and interviews), we can estimate that the fishery has an average production of 30–60 kg per trip, with a potential intermediate value of 45 kg per trip. Another possibility would be that the difference between values reported by fishermen in interviews and those recorded in fish landings one year later reflects a sudden reduction in fish catches. However, we lack temporal data on fish landings to evaluate this hypothesis.

The species diversity in the fishery obtained in the landings is compared with the data from the interviews [35]. The diversity for Paraty reported in the interviews indicates 23 folk species (n = 206) when referring to fishing technology used in the fishery and 63 folk species when referring to fish consumed. In the landings, we found 60 fishery products comprising 57 fish species (Additional file 1). Therefore, the diversity cited by fishers in interviews was very similar to the species richness found in the fishery landings (Additional file 1). These results demonstrate that the fishermen’s information can be helpful when taking an inventory of species, which is important for the conservation of local biodiversity and fishery management. An illustrative example is given by a study [46] based on the information given by local fishermen on the migration and spawning of fifty species in the Mekong river.

### Information useful for managing the paraty fishery

It is important to discriminate the information obtained from the two data collection processes (interviews and systematic sampling at landing points) that could be useful for managing fisheries in Paraty. It is also important to amplify the horizon, connecting the Paraty fishery to other scales or tiers, especially supra and infra tiers. The supra tiers are the ‘Caiçara culture´ (the local culture of the coastal inhabitants of the SE Atlantic Forest coast) and the regional social-economic-ecologic realm in which the Paraty fishery is inserted. The infra tier includes the development of specific understandings of the commercialized and target species that may be affected by fishing. To this end, we paid special attention to one pelagic (king mackerel, *S. cavala*), two estuarine (two species of the genera *Centropomus*), and two reef fish species, *E. marginatus* (Serranidae) and *L. synagris* (Lutjanidae), in our data collection. Considering the data obtained in catch landings and in the macroscopic gonad analysis, we provide the following suggestions regarding the management of the Paraty fishery:

1. Cavala, king mackerel, S. cavalla: sperm was visible in the summer (December to March) (Table 3). The length at first maturity is approximately 50 cm [47]; some homogeneity in length was present in most catches (Figure 5a). King mackerel comprises approximately 1.1% of the catch from the landings (Additional file 1). A suspension of fishing during the summer could benefit this species.

2. Garoupa, dusky grouper, E. marginatus: this reef species, comprising 0.33% of the catch, is very highly prized in the market. Another Serranidae (comb, black and gag grouper, locally called badejo) comprises 0.15% of the landings. The weight-length information reveal that dusky grouper is caught in its early immature stages, mostly 25–40 cm (Figure 5b). Its estimated mean length at first maturity (L50) was 43.8 cm LS for females and 81.3 cm LS for males in the Mediterranean [48]. The results for other nearby artisanal fishing communities on this coast, such as Rio de Janeiro and Bertioga, also demonstrate that these fisheries catch small and immature dusky grouper individuals [37]. As a slow-growth and late-maturing species [48], this fish urgently requires management.

3. Common and fat snook (C. undecimalis and parallelus): common snook is mature in the spring-summer, while fat snook seems to reproduce year-round, according to the macroscopic gonad analysis (Table 3). However, in spring-summer (particularly in the summer), fishermen expend a large amount of effort catching snook. The catch landing results reveal that they catch snook in different size classes (Figures 5d,e). Populations experiencing multiple reproduction periods are less strongly affected than populations that reproduce once a year. Moreover, populations that are affected by fishing at specific age-class levels tend to suffer stronger effects on their population dynamics (such as a tendency to develop smaller individuals due to a fishing target concentrated on large individuals [49]). Fat snook exhibits reproductive periods in Paraty throughout the year; thus, a specific period for restrictive fishing is not strictly necessary. In fact, a suspension of fishing activities at some period in the spring-summer would benefit both snook species (Table 3). Moreover, those species comprise approximately 6% of the total catch in Paraty. Curiously, legislation forbids the fishing of fat snook in the states of Bahia and Espirito Santo (north of Rio on the Brazilian coast) in autumn (March to May) and in Paraná State in November-December (Resolução 060/2009). This legislation does not include the Paraty area and its adjacencies located in the State of Rio [50]. Previous studies of common snook off the coast of Rio and São Paulo have confirmed that spring (September to November) and summer are the periods when eggs are visible [21]. Thus, the legislative suspension of fishing in autumn would not seem applicable for the area of Paraty in the state of Rio de Janeiro.

4. Vermeho ariocó, lane snapper, L. synagris: our study indicates autumn and spring to be important for this species’ reproduction in Paraty (Table 3); however, another study has shown that it reproduces year-round [24]. In that case, any period of restriction in fishing activity could benefit this species. The weight of L. synagris caught by fishermen ranged from 0.1 to 2.5 kg, with a mean of 1.5 kg (N = 29). Compared with previous studies [24], we observe similarities in the weight of the caught individuals of this species, which are mostly small (young individuals).

5. Shrimp: shrimp comprise approximately 35% of the landings in Paraty and are already protected by the defeso system, in which the government pays fishermen when fishing shrimp is forbidden. The defeso is a mechanism for compensating fishermen for not fishing certain species during their respective reproductive periods. This compensation could potentially aid the conservation of biodiversity by conserving stocks. In that regard, compensation functions in a similar manner to some currently observed PES forms (payment for environmental services) [25]. The defeso period for shrimp usually falls in the summer-autumn period.

The *defeso* covers different species of shrimp each year. In the state of Rio de Janeiro, where Paraty is located, *defeso* applies in November-January and in March-May. Regarding fish, snook species are subject to *defeso* in the states of Bahia and Espirito Santo (Portaria 49-N 13/05/1992) in May-July. Other *Epinephelus* (*itajara, niveatus*) fishing is forbidden for 5 years or more, but *E. marginatus*, dusky grouper, is not included [51,52].

To facilitate management, the concentration of the *defeso* in a single period can aid in the administrative and monitoring aspects of management. Considering the ongoing legislation along with the results obtained here, we suggest a summer *defeso* for the fishery in Paraty for the most important target fish species. The summer already includes a *defeso* for shrimp, and this period would include the reproductive periods of important target species such as king mackerel, grouper, snook and lane snapper. In the summer, fishermen benefit from other sources of income, such as conducting trips for tourists. Thus, adding the salary of the *defeso* to tourism earnings could serve as a stimulus for fishermen to restrict their fishing activities in the summer, allowing the stock to recover.

**Figure 6:**
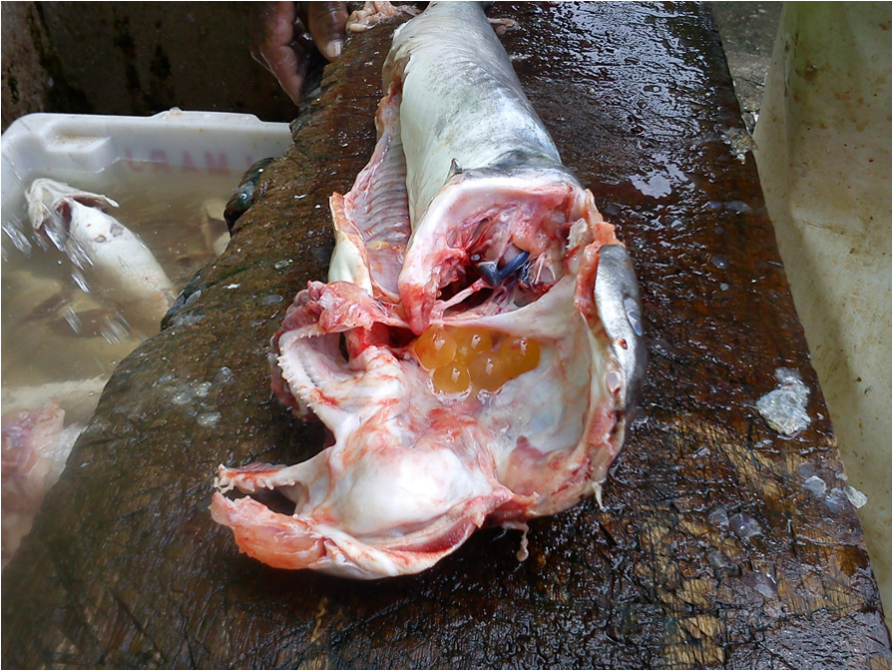
**The catfish bagre cumbaca *Sciades passany*, with mouth brood eggs**

The results from landings also confirmed the importance of other species, such as the sand drum (corvina, *M. furnieri*), xarelete (*Caranx* sp.) and weakfish (*Cynoscion* sp.), which are important commercialized species that account for 11%, 9%, and 4%, respectively, of all of the fish landed (Additional file 1). It is important to conduct research on the reproductive periods of important target fish not studied here, such as the sand drum, weakfish, and catfish. A natural, single observation, made in the field in the fish market in January 2012 by one of the authors (AB) permitted the observation of the catfish locally called bagre cumbaca (*Sciades passany*) with eggs (mouth brooding, Figure 6); thus, the summer period would benefit other species because catfish comprise approximately 3.4% of the landings.

**Figure 7:**
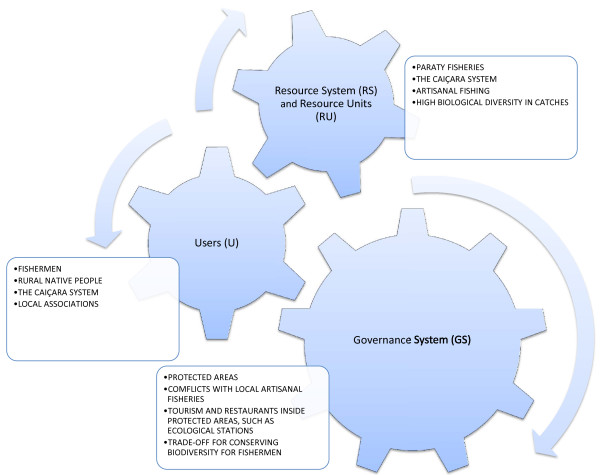
Arrangements of the Caiçara social-ecological system (SES).

The supra tiers of analysis embedding the fishery into the Caiçara system are specifically developed through the SES model (Table 4), which is analyzed in the next section.

**Table 4:** An application of the models of social–ecological systems (SES) to the Paraty fishery

	**Social, Economic, and Political Settings (S)**
**S1- Economic development**	The fishermen from Paraty are rural inhabitants who depend on natural resources for their livelihoods and are part of the Caiçara culture, which includes rural inhabitants of the SE Atlantic Forest coast. Historically, they have been included in the regional and national economic context, shifting their economic activities from small-scale agriculture to fishing and tourism.
**S2- Demographic trends**	Caiçaras are indigenous rural inhabitants who are descendants of Native Indians and Portuguese colonizers. Local populations of Caiçaras have not increased demographically because of outmigration. Nevertheless, the coastal population that is not related to the Caiçaras has increased due to migration from cities such as Rio de Janeiro and São Paulo (tourists and other people linked to services associated with tourism and the environment).
**S3- Political stability**	The local or regional political stability accompanies the political context of the country, which is relatively stable.
**S4- Government settlement policies**	There are conflicts between local inhabitants, the Caiçaras, and the government representatives connected with the protected areas (parks, ecological stations, etc.). These protected areas interfere with the use of resources by the Caiçaras because laws regarding the protection of the environment have forbidden the cultivation of manioc and the production of manioc flour, a basic local staple. Restrictions on fishing in certain areas, particularly on islands, have caused prejudice toward fishermen. Some protected areas more directly affect the fishermen in Paraty, such as Parque Nacional da Bocaina, Estação Ecologica Tamoios, and Área de Proteção Ambiental de Cairuçú.
**S5- Market incentives**	There are programs related to credit for fishing as well as tourism-related activities that increase a fisherman´s earnings. Currently, we have proposed incentives in the form of payments for environmental services (PES) for managing the fisheries in Paraty. Such PES could provide fishermen with a payment to encourage them to help monitor the protected areas and preserve stocks, similar to the defeso system (for details, there is a specific study on PES in Paraty [25]).
**S6- Media organization**	There is no organized information on media information, but the area receives tourism and media incentives. For example, the FLIP, an international literature meeting that is advertised worldwide, is held annually in Paraty; in addition, Paraty aims to become an UNESCO Patrimony.
	**Resource System (RS)**
**RS1- Sector**	Fish
**RS2- Clarity of system boundaries**	There are some mechanisms for the informal division of fishing spots in fishing areas among the coastal communities of Caiçaras, as well as high-level conflicts with intruders from the industrial fisheries in Paraty bay. Boundaries are certainly a very important feature of the resource, and observed boundaries include the boundaries of protected areas as well as those based on the local rules settled by the fishermen, who tend to use spots closer to each of their own communities.
**RS3- Size of resource system**	The size is evaluated based on production from fish catches. In this study, we estimate that artisanal fishing in Paraty, from two landing points, produces an average of 30–60 kg per trip and an annual production of 529,586.40 kg.
**RS4- Human-constructed facilities**	There are local fish stores, markets, restaurants, first-level schools in many communities (there is a high school in the city of Paraty), and a hospital. The local fish market commercializes the resource internally and externally, selling the fish to markets in the city of Rio de Janeiro.
**RS5- Productivity of the system**	Based on the fishermen’s perception (interviews), fish productivity appears to be decreasing for some species; sand drum, shrimp, snook, weakfish, and mullet were frequently mentioned in the interviews.
**RS6- Equilibrium properties**	Equilibrium properties are more difficult to evaluate in unpredictable systems, and a fishery such as Paraty is an uncertain system in which species occur seasonally and there are fluctuations in daily and annual production (see the Additional file 1).
**RS7- Predictability of system dynamics**	Very unpredictable, high variance in production, which can be observed in the Additional file 1 of this study as well as in the literature [33].
**S8- Storage characteristics**	There are fish stores and markets with ice and freezers; however, there are also small-scale fishing communities in Paraty with no electricity except for local power generators or solar energy that function precariously (such as Ponta Grossa and Praia do Sono). Thus, the fish storage capacity varies among the fishing communities, affecting the flow of the local fish market.
**RS9- Location**	High biodiversity tropical areas and areas of fragile domains, such as the Atlantic Forest coast.
	**Governance System (GS)**
**GS1- Government organizations**	In particular, the protected environmental areas created by government.
**GS2- Non-government organizations**	Associations and colônias (Colônias de Pescadores).
**GS3- Network structure**	Fragile, without strong communication channels (compared with the Amazon and with the organization of the fishermen from the neighboring community, Sepetiba Bay).
**GS4- Property-rights systems**	There are systems of informal division of fishing spots among the small-scale communities on the coast, including the fishing communities of Paraty. Nevertheless, property-rights systems in Paraty are incipient and informal because the fishing areas used are close to each of the communities; the fishing spots used in the catches we sampled at landing points confirm such properties. The informal division of fishing areas of small-scale fishermen are not recognized or respected by the industrial fishermen who enter Paraty Bay . Moreover, environmental governmental agencies, forbid artisanal fishers to use spots or to anchor their canoes or boats in islands from protected areas..
**GS5- Operational rules**	Informal acceptance among the artisanal fishing communities of the fishing areas, but no recognition of local rules by other users (industrial fishermen) or by the government (protected areas).
**GS6- Collective-choice rules**	Fishing agreements, payments for environmental services, the defeso system: these are mechanisms that occur in Brazil among fishing communities but not specifically in Paraty (except for the defeso, which is mandated by law).
**GS7- Constitutional rules**	Locally non-observed, only incipient; formally, the defeso from the government (law).
**GS8- Monitoring and sanctioning processes**	These processes are observed, particularly regarding the use of the fishing spots in islands by fishermen when conflicts occur between them and the government agencies.
	**Resource Units (RU)**
**RU1- Resource unit mobility**	Very mobile, but mobility varies among species; some species, such as the cavala (king mackerel), are pelagic and very mobile compared to reef species (groupers and snappers) and invertebrates (shrimp).
**RU2- Growth or replacement rate**	Variable because some species have very slow maturation, such as groupers.
**RU3- Interaction among resource units**	Very interactive, a reasonably strong local knowledge on target species.
**RU4- Economic value**	The economic value of fish and other aquatic organisms is very high because livelihoods depend on these resources. Tourism has been increasing in value for local fishermen, particularly in the summer.
**RU5- Size**	Not estimated, uncertain and highly variable (there are no baseline data that permit an evaluation of stocks of species caught by artisanal fisheries in Paraty (or in Brazil in general).
**RU6- Distinctive markings**	Fishermen identify their catches with distinctive markings. At landing points, fish are often marked to discriminate the catch for commercialization.
**RU7- Spatial & temporal distribution**	Marine organisms are distributed spatially in patches (fish schools, islands with reef fish) and temporally (periods when fish schools pass, periods of growth and reproduction)
	**Users (U)**
**U1- Number of users**	Estimate of the number of artisanal fishers: 485 [35]. The number interviewed in Paraty is 206 artisanal or small-scale fishermen. Other related users are the associations of fishermen and tourists.
**U2- Socioeconomic attributes of users**	Variable among communities because some communities are more isolated than others. Therefore, some communities have higher rates of illiteracy than others, and some are more urban than others. For example, Ponta Grossa in Paraty has a 22% illiteracy rate, compared to 11% in Praia Grande and 5% in Tarituba [35].
**U3- History of use**	Historically, the inhabitants of the Atlantic Forest coast participated in the economic cycles of the region, such as the production of rum from sugar cane. After the decline of this economic activity, local livelihoods depended on small-scale agriculture, particularly the cultivation of manioc and the production of manioc flour, as well as part-time fishing. Agricultural prices declined in the 1950s, and artisanal fishing became the principal economic activity. Currently, both tourism and fishing are part of the economy of these small-scale fishing communities of the Brazilian coast. Local fishermen have thus been associated with tourism, particularly in the summer and during holidays, when they use their boats for tourism activities.
**U4- Location**	Coastal tropical area in the southern Atlantic, Brazil.
**U5- Leadership/entrepreneurship**	Weak, compared to Amazonian artisanal fisheries and other coastal communities on the Atlantic coast.
**U6- Norms/social capital**	Local knowledge is relatively strong and used for fishing, but pressures from protected areas and industrial fisheries, for example, weaken local enterprises.
**U7- Knowledge of SES/mental models**	Local ecological knowledge exists and forms an important category of knowledge for fishery management. This study demonstrates how the two systems of knowledge complement each other and suggests that, in some circumstances, local knowledge could facilitate fishery monitoring.
**U8- Dependence on resource**	Very high.
**U9- Technology used**	Varies from low fishing effort technologies such as hook and lines and set gillnets to other technologies that require increased effort, such as small trawlers used to catch shrimp and the “cerco do robalo”, a method used in the community of Tarituba, among other local communities, to capture snook with dubious ecological soundness but with good economic returns.
	**Interactions (I) Outcomes (O)**
**I1- Harvesting levels of diverse users**	Artisanal fisheries conflict with industrial fishermen that enter the bay. Artisanal fishermen in Paraty also use a diverse array of techniques to fish. A solution to manage the fishery at Paraty is to separate users and fishing technologies and utilize different approaches for each with respect to management necessities and intentions. Certainly, the exclusion of industrial fishermen from Paraty bay is necessary and required by coastal legislation, which is not followed as it should be, causing conflicts between small-scale and industrial fishermen [25].
**I2- Information sharing among users**	Still weak compared to other fishing areas of the coast and Amazonian fisheries; it can be strengthened by cooperation with other organized fishermen, such as those from Ilha Grande and Sepetiba Bay.
**I3- Deliberation processes**	Non-explicit, variable, data not organized or inaccessible.
**I4- Conflicts among users**	High conflict between artisanal and industrial fishermen and between artisanal fishermen and environmental governmental agencies. Industrial fisheries enter fishing spots and the bay, causing conflicts over the use of the aquatic space.
**I5- Investment activities**	A very tourism-heavy area in which investments occur. International meetings are held in Paraty, such as the International literary meeting (the FLIP), as well as other tourism-associated investments. There is international tourism year-round in Paraty.
**I6- Lobbying activities**	Data not available or not systematically organized or accessible.
**O1- Social performance measures**	Efficiency (a measure of economic returns in catches) and equity (a measure of social distribution) are important aspects to follow in Paraty fisheries, and it is possible to address these variables for future suggestions for fishery management.
**O2- Ecological performance measures**	Catch diversity is an important measure that can be addressed, as shown in this study. We observed more than 50 organisms in fish catches, as shown in the Additional file 1 of this study. The high diversity of small-scale or artisanal fishermen is a source of resilience because there can be some substitutability of target species. Resilience could also be addressed through other variables, such as a) economic returns of catches; b) perceptions of fishermen on the abundance of the resource; c) management rules in fishing, particularly for reef fishing; and d) substitutability of activities, taking into consideration that tourism is an economic alternative.
**O3- Externalities to other SESs**	In this case, externalities from the fishery system affect the conservation of biodiversity (in protected areas). Protected areas include externalities that affect the Paraty fishery and the fishermen’s earnings.
	**Related Ecosystems (ECO)**
**ECO1- Climate patterns**	Tropical climate, with a rainy season (summer) and a dry season (winter).
**ECO2- Pollution patterns**	The Paraty coast, in particular, receives organic discharges from domestic sewage. Paraty bay t is located adjacent to the two nuclear power plants in Brazil, as well as to Sepetiba bay, a highly contaminated bay that received industrial discharges, which are polluted with heavy metals and sewage. At Paraty, there are several small harbors, particularly for tourists (marinas), and shipyards.
**ECO3- Flows into and out of the focal SES**	Connection through different scales and tiers with sets of variables permits the interconnection of Paraty fisheries with the Caiçara system and culture as well as with the economic and ecological systems of the region The SES model is a mechanism by which data and variables can be linked into a more general system. In that regard, a trade-off analysis that considers the drivers for biodiversity conservation (that affects fishermen and tourists) and the economic temptation of the fishermen to increase catches and earnings can be visualized through the variables shown in this table. Through the SES model, the frontiers or thresholds in the decision-making processes of fishermen can be associated with different tiers, including an infra tier of when to fish a target species through a supra tier, such as the Caiçara system and its ecological-economic constraints. Figure 7 illustrates this analysis, and the links among tiers. SES could also be useful for understanding temporal shifts and flows: for example, the history of the use of the resource shows how economic pressure through price changes moved the Caiçaras from agriculture (and part-time fishermen) to full-time fishermen. Currently, tourism plays an important role by pushing fishermen to allocate time to this activity, thus causing some fishermen to assume a part-time role in a small-scale fishery.

### Paraty fishery management within the Caiçara system-fishermen´s perceptions (interviews), catches (landings) and data integration through SES (Social-Ecological Systems)

The striking diversity of artisanal fisheries forms a basis for their sustainability. This diversity is observed from interviews and catch landings and is in agreement with the two systems of knowledge (Table 1 in Additional file 1). The users’ knowledge of the resource, in this case that of the fishermen, serves as a basis for the diagnosis of fishery behavior. Using systematic data collection at landing points, we could complement and analyze information derived from the different systems of knowledge. Our analysis now decomposes the variables and re-integrates them to propose a management policy for the Paraty fishery.

Table 4 is based on the use of the SES model [34]. The application of the SES model can be seen as a protocol that aids in the decomposition of the system into a set of related variables; as suggested [34], SES model aids in connecting variables and analyzing the complexity of the system (in this study, the Paraty artisanal fishery). Fisheries are complex. Part of this complexity is derived from catch uncertainty because species and biomass vary between fishing trips, days and seasons. In Table 4, we show the SES of the Paraty fishery, describing the resource system (a fishery), the resource unit (fish or aquatic organisms, such as crustaceans), the users (artisanal fishermen and their families) and the governance (local rules and governmental strategies, such as protected areas). The set of variables connected through different tiers permits the analysis of their interconnections and, consequently, an understanding of the Paraty fishery SES integrated with its history and its ecologic-cultural-socio-economic context.

### Concluding remarks

The Paraty fishery needs management. Some target species are caught at immature or early ages. To be successful, management of the Paraty fishery should be integrated at different levels and in its own regional market and culture. In this regard, the SES model facilitates an understanding of the following aspects:

(1) Fishing was not historically a full-time activity. As part of the Caiçara culture, fishing was a part-time activity, although this trend began to shift toward full-time fishing in the 1950s. Currently, tourism supplements the earnings of the fishermen, and fishermen interact with tourists on holidays and in the summer.

(2) Periods in which fishing is not allowed are necessary for some species. In that regard, the use of a current mechanism, the defeso, can be an important motivator for fishermen to help maintain fish stocks [25].

**Figure 8:**
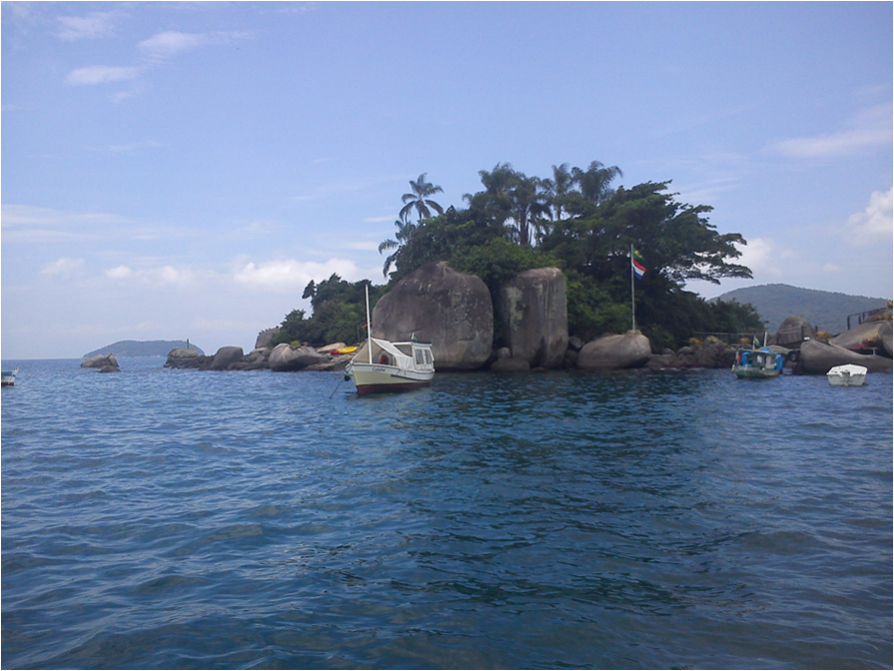
The Ilha do Catimbau, anchored boats and restaurant inside the Ecological Station of Tamoios, RJ, Brazil.

In particular, some aspects should be highlighted to more fully understand the complexity of this system. The major conflicts of fishermen are 1) the industrial fishing boats that enter the bay; 2) the protected areas that prevent fishermen from utilizing some islands of the bay; and 3) discrepancies or discrimination on the part of the monitoring authorities, which permit restaurant owners and other properties inside the protected areas, in contrast to the inflexible behavior that is directed against small-scale or artisanal fishermen, such as not allowing them to fish or even stop their boats at islands of the Ecological Station, a restricted protected area [25,35]. In that regard, it is illustrative to observe (Figure 8) anchored boats and to mention a restaurant in the Ilha do Catimbau, a protected area (the restrictive Tamoios Ecological Station). For the first time since we began studying Paraty in 2009, we have learned that governmental environmental agencies, represented by INEA, taxed island users (report in May 2, 2012, INEA, http://www.inea.rj.gov.br).

SES models permit the discrimination of variables related to the Paraty artisanal fishery and their links to the social-ecological system and, in particular, to tiers at different scales, as suggested elsewhere [34]. In that regard, it is possible to select and prioritize sub-systems and their interactions (Figure 7). The major subjects of Figure 7 are detailed in Table 4. Through this careful analysis, we can better evaluate the trade-off fishermen consider when navigating different activities (from fishing to tourism), accepting the importance of biodiversity conservation, or both. We recommend stressing the following aspects for conserving biodiversity and obtaining a sustainable artisanal fishery in Paraty.

a) There is no ‘ecologically noble savage’: there are examples of non-conserving behavior by small-scale societies, such as habitat degradation and faunal extinction [56]. In this study, we observed some fish caught at early ages and an ecologically questionable technique for catching snook. The tendency to consider folk knowledge itself as an example for conservation has been criticized by some authors [57]. Even when there is significant local or folk knowledge, it is occasionally inaccurate [20] or restricted to a target species [24]. There are, however, examples of folk knowledge that includes conservation practices [5, 9, 56]. Among the Caiçaras or artisanal fishermen of the SE coast of Brazil, there are informal local rules on the division of fishing spots that can be seen as incipient forms of management [23].

b) The landings from the Paraty fishery include a high diversity of species, typical of a small-scale tropical fishery, where a wide array of gear and techniques are used to obtain a high biological diversity of organisms. Such diversity brings food security to individuals, whose consumption is highly diverse in terms of fish and to the market, which also desires a high diversity of options for consumers and can accommodate the seasonality of supply.

c) Drivers and stimuli for conserving biodiversity: one of the problems of perceiving local knowledge as enhancing biodiversity conservation is that a simple failure to overharvest is considered or taken as an indicator of conservation [56]. This perception is actually a misinterpretation of a conservative behavior. For example, resources may be maintained in an area due to a small population of extractors or users and not due to an intention to conserve biodiversity per se. In that sense, it is important to define behaviors that will be de facto directed toward biodiversity conservation. An obstacle for rural native people in the SE coast of Brazil is that their livelihoods are dependent on natural resources [35]. Here, where fishermen need to market the natural or raw products, what are the incentives for conserving biodiversity? From a fisherman´s perception, “Why not fish if I need to fish to sustain my family?” This obstacle highlights the need for short-term compensation [25] because delayed benefits are questionable.

d) Delayed benefit: this aspect is one of the most important because it implies decision-processes related to co-partnership or co-management towards conservation. Conservation practices, such as restraints on harvesting or extracting resources, can be costly in the short term [25, 56]. The trade-off between present restraints and future gains raises the issue of temporal discounting [56]. The trade-off of restraining fishing today for a better livelihood tomorrow is not obvious to fishermen [25]. Without a payment to compensate for the loss of earnings from fishing, it is probably impossible to involve local fishermen in the conservation of fish stocks. The defeso, or a payment for an environmental service [25], would be one incentive for fishermen to restrict fishing.

e) Moving from fishing to tourism: this transition would be a second economic benefit to fishermen and an ecological gain for the conservation of fish but would result in a decrease in the diversity of food consumed. Small-scale fisheries are responsible for delivering a high diversity of fish to consumers. This study illustrated such diversity through catch landings and interviews with fishermen. Industrial fishing concentrates on a few species, such as sardines, tuna, small sharks, and croakers. Therefore, tourism may draw fishermen away from fishing, but it does not solve the food security concern (for maintaining the diversity of food consumed and commercialized). In other words, tourism is a complementary and very adequate source of income for fishermen because it can increase their standard of living, but it is not a management strategy for the fishery in stricto sensu.

Considering path dependency and mechanisms of feedback between the past and future [13], we perceive that, despite the existence of historical local rules on fishing areas concerning the use of local resources, the governmental authorities did not specifically consider these rules for the management of protected areas in the region where fishing occurs. Therefore, there is no dialectic process, a back-and-forth process between local knowledge and governmental criteria, in this case. These obstacles function as an impediment for management to proceed in the area. Therefore, alternative incentives are needed, such as those in the form of payments (PES), to overcome the losses that fishermen suffer if they direct their efforts to the conservation of fish stocks.

## Competing interests

The authors declare that they have no competing interests.

## Authors’ contribution

AB idealized the project on fish landings, coordinated the study, collected, organized, analyzed data, and wrote the manuscript; SS organized and analyzed the data, as well as have proceed with statistical analysis, where needed; VN collected the data an contributed with ideas on the study and discussion; PL and RS contributed with ideas and in the discussion. All authors read and approved the final manuscript.

## Supplementary Material

Additional file 1 Supplementary Material Begossi el al. Click here for file
