# Supplementary material for: The paraty artisanal fishery (southeastern Brazilian coast): ethnoecology and management of a social-ecological system (SES)
Source: J Ethnobiol Ethnomed. 2012 Jun 27;8:22. doi: 10.1186/1746-4269-8-22 (PMC3476967; doi:10.1186/1746-4269-8-22)
Supplement: Additional file 1 — Supplementary Material Begossi el al. [file 1746-4269-8-22-S1.docx]

SUPLEM. MATERIAL

Begossi el al

Appendix


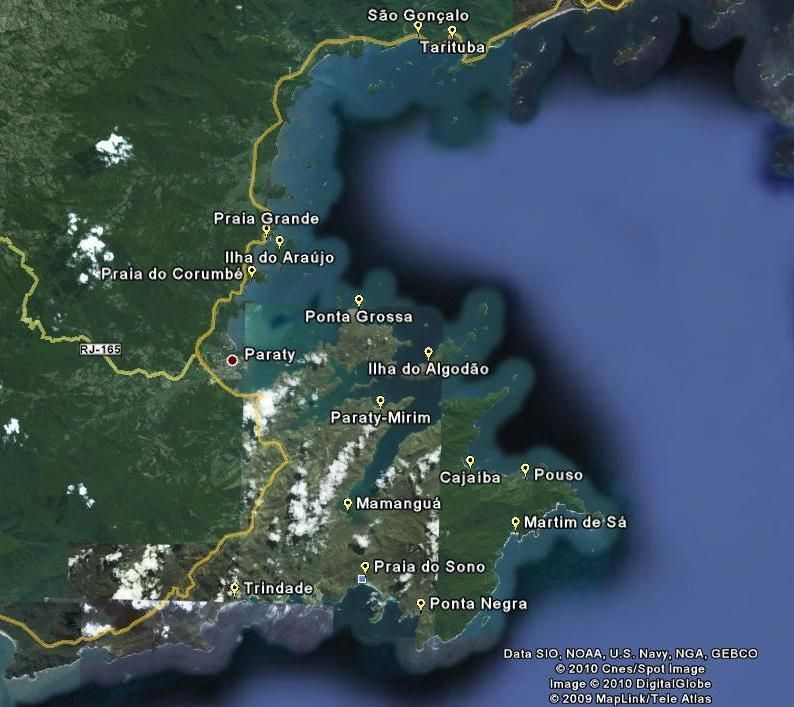


**Paraty bay**

Figure 1 (source: <http://umanitoba.ca/institutes/natural_resources/Brazil/index.html>)


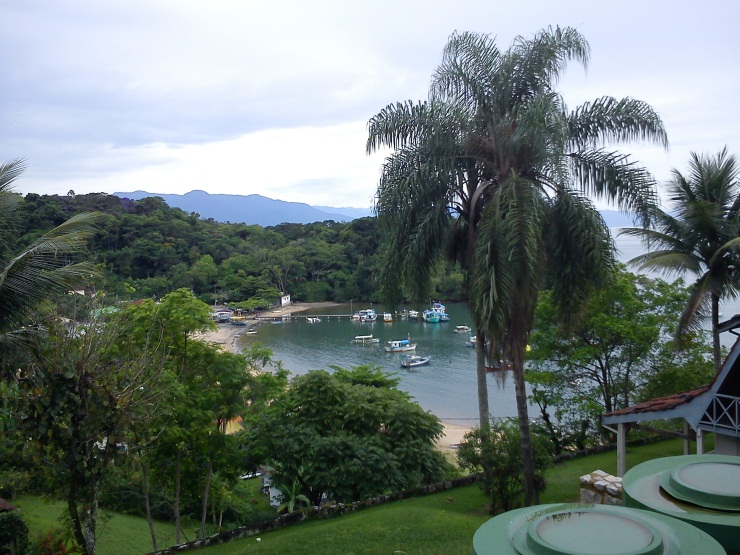


Figure 2a. General view of Praia Grande, Paraty, 2012


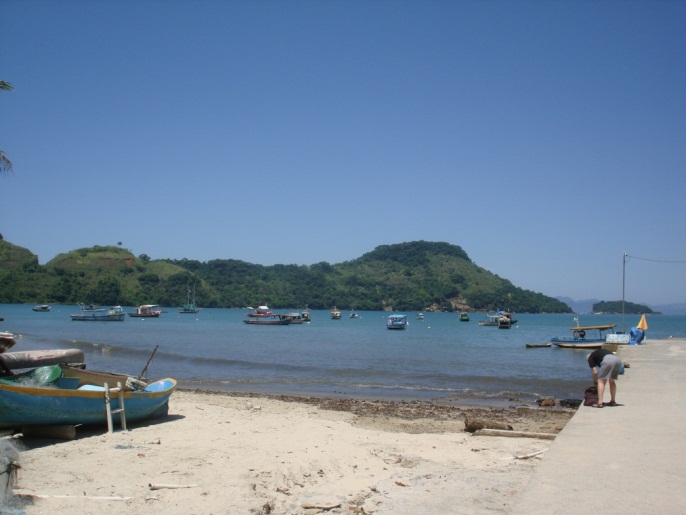


Figure 2b. Tarituba (landing point)

Table 1. Sample of production (kg) (November 2009- December 2010) in Paraty at the landing points of Praia Grande and Tarituba

| ***Species*** | **Value (kg)** | | | **%** | | |
| --- | --- | --- | --- | --- | --- | --- |
|  | Pr. Grande | Tarituba | Total | Pr. Grande | Tarituba | Total |
| Camarão | 2,927.00 | 1,145.90 | 4,072.90 | 40.52 | 26.97 | 35.50 |
| Corvina | 943.50 | 366.40 | 1,309.90 | 13.06 | 8.62 | 11.42 |
| Xerelete |  | 1,020.00 | 1,020.00 | 0 | 24.00 | 8.89 |
| Pescada | 466.85 | 48.60 | 515.45 | 6.46 | 1.14 | 4.49 |
| Robalo-flecha | 381.45 | 103.85 | 485.30 | 5.28 | 2.44 | 4.23 |
| Tainha | 439.50 | 15.50 | 455.00 | 6.08 | 0.36 | 3.97 |
| Paraty | 408.90 | 23.90 | 432.80 | 5.66 | 0.56 | 3.77 |
| Bagre | 228.60 | 157.30 | 385.90 | 3.16 | 3.70 | 3.36 |
| Prejereba | 277.30 |  | 277.30 | 3.84 | 0 | 2.42 |
| Bonito | 128.50 | 122.00 | 250.50 | 1.78 | 2.87 | 2.18 |
| Cação | 146.10 | 52.50 | 198.60 | 2.02 | 1.24 | 1.73 |
| Lula | 153.00 | 33.20 | 186.20 | 2.12 | 0.78 | 1.62 |
| Paranaguaiu |  | 182.00 | 182.00 | 0 | 4.28 | 1.59 |
| Mistura | 92.20 | 83.50 | 175.70 | 1.28 | 1.96 | 1.53 |
| Olho de cão | 105.00 | 65.50 | 170.50 | 1.45 | 1.54 | 1.49 |
| Sororoca | 62.45 | 92.70 | 155.15 | 0.86 | 2.18 | 1.35 |
| Robalo Peba | 106.40 | 30.38 | 136.78 | 1.47 | 0.71 | 1.19 |
| Arraia | 110.80 | 15.00 | 125.80 | 1.53 | 0.35 | 1.10 |
| Cavala | 17.14 | 107.13 | 124.27 | 0.24 | 2.52 | 1.08 |
| Vermelho | 53.00 | 32.05 | 85.05 | 0.73 | 0.75 | 0.74 |
| Galo | 5.60 | 69.60 | 75.20 | 0.09 | 1.64 | 0.67 |
| Bicuda | 1.60 | 61.80 | 63.40 | 0.02 | 1.45 | 0.55 |
| Cocoroca |  | 62.00 | 62.00 | 0 | 1.46 | 0.54 |
| Espada | 29.60 | 24.00 | 53.60 | 0.41 | 0.56 | 0.47 |
| Sardinha | 11.40 | 41.43 | 52.83 | 0.16 | 0.97 | 0.46 |
| Carapau |  | 47.50 | 47.50 | 0 | 1.12 | 0.41 |
| Piranjica | 3.60 | 42.00 | 45.60 | 0.05 | 0.99 | 0.40 |
| Guivira | 32.30 | 9.60 | 41.90 | 0.45 | 0.23 | 0.36 |
| Garoupa | 15.39 | 22.75 | 38.14 | 0.21 | 0.53 | 0.33 |
| Porquinho |  | 32.00 | 32.00 | 0 | 0.75 | 0.28 |
| Coroco |  | 30.00 | 30.00 | 0 | 0.71 | 0.26 |
| Jaguruça |  | 24.00 | 24.00 | 0 | 0.56 | 0.21 |
| Sambalo |  | 19.50 | 19.50 | 0 | 0.46 | 0.17 |
| Badejo | 14.67 | 2.70 | 17.37 | 0.20 | 0.06 | 0.15 |
| Dourado |  | 15.00 | 15.00 | 0 | 0.35 | 0.13 |
| Marimbá |  | 14.50 | 14.50 | 0 | 0.34 | 0.13 |
| Sargo de Beiça | 8.00 | 5.10 | 13.10 | 0.11 | 0.12 | 0.11 |
| Caçonete | 13.00 |  | 13.00 | 0.18 | 0 | 0.11 |
| Ubeba | 11.50 | 1.20 | 12.70 | 0.16 | 0.03 | 0.11 |
| Guaiá | 10.00 |  | 10.00 | 0.14 | 0 | 0.09 |
| Xaréu |  | 7.00 | 7.00 | 0 | 0.16 | 0.06 |
| Albarana(Ubarana) | 5.00 | 2.00 | 7.00 | 0.07 | 0.05 | 0.06 |
| Linguado | 2.40 | 3.50 | 5.90 | 0.03 | 0.082 | 0.05 |
| Baiacu | 5.60 |  | 5.60 | 0.09 | 0 | 0.05 |
| Roncador |  | 5.00 | 5.00 | 0 | 0.118 | 0.04 |
| Siri Azul |  | 3.20 | 3.20 | 0 | 0.075 | 0.03 |
| Moreia |  | 3.00 | 3.00 | 0 | 0.071 | 0.03 |
| Rêmora | 2.20 |  | 2.20 | 0.03 | 0 | 0.02 |
| Companheiro | 2.00 |  | 2.00 | 0.03 | 0 | 0.02 |
| Paru |  | 1.50 | 1.50 | 0 | 0.035 | 0.01 |
| Betara | 0.80 |  | 0.80 | 0.01 | 0 | 0.01 |
| Samabari |  | 0.70 | 0.70 | 0 | 0.02 | 0.01 |
| Maria Luiza |  | 0.60 | 0.60 | 0 | 0.01 | 0.005 |
| Salema |  | 0.50 | 0.50 | 0 | 0.01 | 0.004 |
| Cangoá Mulata | 0.20 |  | 0.20 | 0.003 | 0 | 0.002 |
| João cachaça | 0.15 |  | 0.15 | 0.002 | 0 | 0.001 |
| Derretida | 0.10 |  | 0.10 | 0.001 | 0 | 0.001 |
| *All* | *7,222.80* | *4,249.09* | *11,471.89* | *100* | *100* | *100* |

Figure 3. Comparisons of the catch in December of 2009 and 2010.

Table 2. Selected fish identification (from Praia Grande, Paraty, RJ)

| **Family** | **Local name** | **Species** |
| --- | --- | --- |
|  |  |  |
| **Ariidae** | bagre amarelo | *Aspistor luniscutis* |
|  | bagre cumbaca | *Sciades passany* |
| **Carangidae** | xaréu, xaréu-branco | *Alectis ciliaris* |
|  | carapau | *Caranx crysos* |
|  | xerelete | *Caranx ruber* |
|  | guaivira | *Oligoplites saliens* |
|  | pampo | *Trachinotus carolinus* |
| **Carcharhinidae** | cação rato | *Carcharhinus porosus* |
| **Centropomidae** | robalo-peva, robalo-peba, cambira | *Centropomus parallelus* |
|  | robalo flecha | *Centropomus undecimalis* |
| **Coryphaenidae** | dourado | *Coryphaena hippurus* |
| **Ephippididae** | paru | *Chaetodipterus faber* |
| **Gempylidae** | serra | *Thyrsitops lepidopoides* |
| **Haemulidae** | sargo | *Anisotremus surinamensis* |
| **Holocentridae** | mangorra | *Holocentrus ascencionis* |
| **Kyphosidae** | piragica | *Kyphosus* sp. |
| **Lobotidae** | prejereba | *Lobotes surinamensis* |
| **Lutjanidae** | vermelho, cióba verdadeiro | *Lutjanus synagris* |
| **Mugilidade** | parati, parati olho de fogo | *Mugil gaimardianus* |
|  | tainha | *Mugil liza* |
| **Priacanthidae** | olho de cão | *Priacanthus arenatus* |
| **Sciaenidae** | goete, pescadinha | *Cynoscion jamaicensis* |
|  | pescada bicuda ou cambuçú | *Cynoscion jamaicensis* |
|  | pescada branca | *Cynoscion leiarchus* |
|  | ubeba | *Larimus breviceps* |
|  | pescada banana | *Macrodon ancylodon* |
|  | corvina | *Micropogonias furnieri* |
|  | pescada azeitona | *Nebris microps* |
|  | corvina amarela | *Ophioscion punctatissimus* |
| **Scombridae** | sororoca | *Scomberomorus brasiliensis* |
|  | bonito, bonito cadelão | *Auxis thazard* |
|  | cavala | *Scomberomorus cavalla* |
| **Serranidae** | garoupa legitima | *Epinephelus marginatus* |
|  | badejo | *Mycteroperca bonaci* |
|  | badejo da areia | *Mycteroperca microlepis* |
| **Xiphiidae** | agulha | *Xiphias gladius* |
